# Serum uric acid and non-alcoholic fatty liver disease in non-obesity Chinese adults

**DOI:** 10.1186/s12944-017-0531-5

**Published:** 2017-10-16

**Authors:** Xiaoya Zheng, Lilin Gong, Rong Luo, Hua Chen, Bin Peng, Wei Ren, Yonghong Wang

**Affiliations:** 1 Department of Endocrinology, the First Affiliated Hospital of Chongqing Medical University, 1 Friendship Road, Yuzhong District, Chongqing, NO China; 2 The Public Health Center, the First Affiliated Hospital of Chongqing Medical University, 1 Friendship Road, Yuzhong District, Chongqing, NO China; 3 Department of Statistics, Chongqing Medical University, Chongqing, China

**Keywords:** Nonalcoholic fatty liver disease, Chinese, Uric acid, Non-obesity, Ultrasonography

## Abstract

**Background:**

Previous studies found elevated serum uric acid (SUA) was associated with the development or progression of non-alcoholic fatty liver disease (NAFLD) in general population; in this study we aim to investigate the association of SUA and the severity of NAFLD based on grade of fatty liver on ultrasonography in non-obese subjects.

**Methods:**

Data were obtained from subjects via routine physical examinations in the Public Health Center of our hospital between 2011 and 2014. The data included completed anthropometry and blood biochemical indicators and the results of abdominal ultrasound. The diagnosis of NAFLD was according to the clinical diagnosis of the Guidelines for the diagnosis and treatment of nonalcoholic fatty liver disease in 2008.

**Results:**

In total, 95,924 subjects were analyzed in this study. The prevalence rate of lean-NAFLD was 8.16%, among which 7.58% had mild steatosis, and 0.58% had moderate and severe steatosis. The prevalence of fatty liver was increased progressively with SUA. Among which the prevalence of mild fatty liver from Q1 to Q4 were 10.33%, 18.39%, 23.11% and 25.93%; the prevalence of moderate and severe fatty liver from Q1 to Q4 were 1.06%, 2.82%, 5.05% and 7.27%. Lean-subjects with hyperuricemia had an OR of 1.718 (95% CI 1.622–1.820) to have NAFLD, after adjusted for other metabolic disorders. The area under curve (AUC) for detecting mild fatty liver based on SUA was 0.70; and the AUC for detecting moderate and severe fatty liver based on SUA was 0.78.

**Conclusions:**

Our data showed positive associations between elevated SUA levels and lean-NAFLD risk in the inland Chinese adults, independent of other metabolic factors. Our study also suggests that SUA could be considered as a simple and non-invasive method to follow up patients with lean-NAFLD.

## Background

Non-alcoholic fatty liver disease (NAFLD) has become an important public health issue because of its high prevalence [1]. It has been estimated that its prevalence varies between 20 and 30% in western countries, and 15–30% of the general population in China [2]. NAFLD is commonly associated with obesity, type 2 diabetes mellitus, dyslipidemia and hypertension, and has also been regarded as a hepatic manifestation of metabolic syndrome (MetS) [3].

Similar to NAFLD, serum uric acid (SUA), the end product of purine metabolism by liver, also has been linked to both MetS and cardiovascular disease [4]. Recently, several evidences suggest that elevated SUA frequently associates with the development or progression of NAFLD [5–8]. Prior epidemiological study [9] showed that SUA is an independent risk factor for cardiovascular diseases, and the process included insulin resistance, which is considered as an important risk factor for the development of NAFLD. A recent study [10] demonstrated that elevated uric acid level is independently associated with ultrasound-diagnosed NAFLD, regardless of insulin resistance.

Obesity is an important risk factor for NAFLD. However, NAFLD can also occur in non-obese subjects. The prevalence of NAFLD varied from 15% to 21% in non-obese Asians [body mass index (BMI) < 25] [11]. NAFLD can be considered as an early predictor of metabolic disorders and a major cause of cryptogenic liver disease in normal-weight population. Chinese people have lower BMI than people in western countries, but a similar prevalence of NAFLD. There might be different metabolic features in Chinese adults [12]. We conducted this study to characterize metabolic characters of non-obese-NAFLD and identify its association with SUA.

For definite diagnosis and staging of NAFLD, liver biopsy remains the gold standard. Liver biopsy, as an invasive approach plus its practical difficulties and high expenses further justify the need for a safe and reliable approach for follow-up of patients with NAFLD [13]. So, in present study, we also intended to investigate the association between SUA and the severity of NAFLD based on grade of fatty liver on ultrasonography.

## Data and methods

Data were obtained from subjects who underwent routine health examination in the Public Health Center of the First Affiliated Hospital of Chongqing Medical University from January 2011 through December 2014. Exclusion analysis criteria:1) Age<18y or age>80y; 2) BMI>25 Kg/m^2^;3) Alcohol consumption>140 g/week for men and 70 g/week for women; 4) Subjects taking antihypertensive agents (especially diuretics, such as furosemide), anti-diabetic agents, lipid-lowing agents, or hypouricemic agents (including drugs that inhibit uric acid synthesis, such as allopurinol, and drugs that promote uric acid excretion, such as sulfinpyrazone, probenecid and benzbromarone); 5) other known chronic liver diseases such as viral hepatitis or autoimmune hepatitis. 6) Subjects with incomplete data involving ualtrasonography; blood test values were excluded for analyzing to avoid bias. The study was approved by Ethics and Human Subject Committee of Chongqing Medical University. Given the retrospective nature of the study, we were granted a waiver of informed consent.

Anthropometric measurements included height, weight, waist circumference, and blood pressure. Height and weight were measured while the subjects were wearing light clothing and no shoes. BMI was calculated as weight (kg) divided by the square of the height (m). Waist circumference was measured at the midpoint between the bottom of the rib cage and the top of the iliac crest at the end of exhalation. Blood pressure, including systolic blood pressure (SBP) and diastolic blood pressure (DBP), was measured using each subject’s right arm after a 5 min rest and in a sitting position. Blood biochemical analyses included serum uric acid (SUA), cholesterol (TC), triglycerides (TG), low-density lipoprotein cholesterol (LDL-c), high-density lipoprotein cholesterol (HDL-c), fasting plasma glucose (FPG), alanine aminotransferase (ALT), and aminotransferase (AST). These indicators were measured by an inmmunochemical-automated analyzer (Type 7600, Hitachi Ltd., and Japan). All participants agreed to abdominal B-ultrasonography examinations.

Abdominal ultrasonography was performed by experienced technicians on HD7 ultrasound system (Philips, Shenyang, China). The diagnosis of NAFLD was according to the clinical diagnosis of the Guidelines for the diagnosis and treatment of nonalcoholic fatty liver disease in 2008. In addition, the ultrasonography results were differentiated into mild, moderate and severe cases of fatty liver according to the ultrasonic diagnosis as per guidelines [14].

### Statistical analyses.

Statistical analyses were performed using SAS software (version 9.0; SAS Institute, Inc., Cary, North Carolina). Continuous variables with normal distributions are expressed as the mean ± standard deviation ($\overline{X}$±SD), and categorical variables are described as percentages (%). Multiple-group comparisons of means were performed using generalized linear models (GLMs). Chi-square tests were used to compare percentages. Logistic regression was used to obtain odds ratios (ORs) of categorical variables. Multi-factor logistic regression analysis was used to control for age, sex, waist, and so on. The diagnostic performance of the SUA was assessed using the receiver operating characteristic (ROC) curve. All statistical analyses were two-sided, and *p*-values less than 0.05 were considered to be statistically significant.

## Results

In total, 95,924 subjects were analyzed in this study. A total of 84,421 non-obesity participants (91.84%) did not have NAFLD, 7503 non-obesity participants (8.16%) had NAFLD, among which 6967 participants (7.58%) had mild steatosis, and 536(0.58%) had moderate and severe steatosis. NAFLD had a male predominance. Mild fatty liver in the male accounted for 77.22%, while moderate and severe fatty liver in the male accounted for 86.61%. After adjusting for age, BMI and waist, the SBP, DBP, TC, TG, LDL-c, ALT, and SUA were significantly higher in patients with NAFLD than in controls. Only HDL-c was lower in patients with NAFLD than in controls. Among the patients with NAFLD, the patients with moderate and severe fatty liver tended to have higher SBP, DBP, TC, TG, LDL-c, ALT, and SUA, and lower age and HDL-c compared with mild fatty liver patients. Details are shown in Table 1.

**Table 1:** Demographic and laboratory variables of the studied adults

N (%)	Non-NAFLD 84421 (91.84)	NAFLD 7503 (8.16)		*P*-value
		Mild 6967 (7.58)	Moderate and severe 536 (0.58)	
Male (%)	49.31	77.22	86.61	< 0.001
Age(y)	42.94 ± 14.44	47.27 ± 1.46	44.98 ± 12.82	< 0.001
BMI (Kg/m^2^)	23.43 ± 1.27	23.94 ± 0.91	24.08 ± 0.81	< 0.001
Waist (cm)	77.11 ± 8.37	87.30 ± 7.30	91.85 ± 7.50	< 0.001
SBP (mmHg)	118.85 ± 18.07	130.58 ± 18.86^a^	134.73 ± 17.99^b^	< 0.001
DBP (mmHg)	73.12 ± 11.47	80.79 ± 12.01^a^	84.68 ± 12.19^b^	< 0.001
TC (mmol/L)	4.77 ± 0.90	5.14 ± 0.98^a^	5.24 ± 1.01^b^	< 0.001
TG (mmol/L)	1.29 ± 0.98	2.35 ± 1.99^a^	2.96 ± 2.49^b^	< 0.001
HDL-c(mmol/L)	1.53 ± 0.37	1.26 ± 0.31^a^	1.17 ± 0.28^b^	< 0.001
LDL-c(mmol/L)	2.77 ± 0.78	3.11 ± 0.82^a^	3.15 ± 0.84^b^	< 0.001
ALT(U/L)	16.00 (12,23)	26 (19,38) ^a^	39 (27,58) ^b^	< 0.001
AST(U/L)	20.00 (17,24)	23 (19,28)	27 (22,35)	< 0.001
SUA (μmol/L)	318.70 ± 80.52	378.79 ± 87.80^a^	409.08 ± 92.30^b^	< 0.001
Male	378.5 ± 75.5	411.95 ± 83.71^a^	439.43 ± 89.13^b^	< 0.001
Female	278.11 ± 58.74	320.59 ± 70.30^a^	350.26 ± 82.18^b^	< 0.001

*Abbreviations: ALT* alanine aminotransferase, *AST* aspartate aminotransferase, *BMI* body mass index, *DBP* diastolic blood pressure, *HDL-c* high density lipoprotein-cholesterol, *LDL-c* low-density lipoprotein-cholesterol, *SBP* systolic blood pressure, *SUA* serum uric acid, *TC* total cholesterol, *TG* triacylglycerol

^a^ means *P* < 0.05 compared with Non-NAFLD, after adjusted for age, BMI, and waist

^b^ means *P* < 0.05 compared with mild fatty liver, after adjusted for age, BMI, and waist

**Fig. 1:**
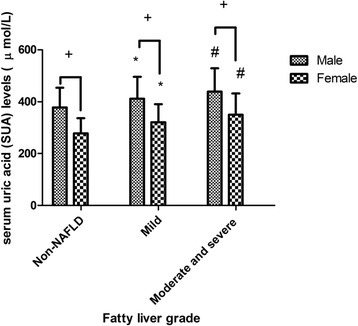
Serum uric acid levels of male and female patients with mild fatty liver compared to those with moderate and severe fatty liver. ^+^ means *P* < 0.05 compared with female. * means *P* < 0.05 compared with Non-NAFLD. ^#^ means *P* < 0.05 compared with mild fatty liver

The SUA levels in male were significantly higher than in female either with or without fatty liver. Patients with fatty liver had significantly higher levels of SUA than subjects without fatty liver, while patients with moderate and severe fatty liver had significantly higher levels of SUA than patients with mild fatty liver both in males and females (Fig. 1).

**Fig. 2:**
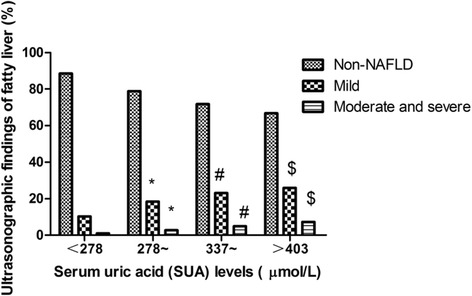
Ultrasonographic findings of fatty liver according to quartile (Q) of serum uric acid (SUA). Q1: <278 mmol/L, Q2: 278–336 mmol/L, Q3: 337–403 mmol/L and Q4 > 403 mmol/L. ^*^ means *P* < 0.05 compared with Q1; ^#^ means *P* < 0.05 compared with Q2; ^$^ means *P* < 0.05 compared with Q3

Quartiles according to SUA in this population were categorized as follows: Q1: <278 mmol/L, Q2: 278–336 mmol/L, Q3: 337–403 mmol/L and Q4 > 403 mmol/L. The prevalence of fatty liver was increased progressively with SUA. Among which the prevalence of mild fatty liver from Q1 to Q4 were 10.33%, 18.39%, 23.11% and 25.93%; the prevalence of moderate and severe fatty liver from Q1 to Q4 were 1.06%, 2.82%, 5.05% and 7.27% (Fig. 2).

**Fig. 3:**
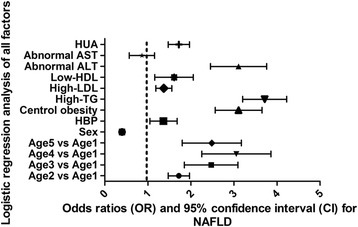
Logistic regression analysis of all risk factors of NAFLD. Abbreviations: ALT, alanine aminotransferase; AST, aspartate aminotransferase; HBP, High blood pressure; HDL, high density lipoprotein; HUA, hyperuricemia; LDL, low-density lipoprotein; SBP, systolic blood pressure; SUA, serum uric acid; TG, triacylglycerol. NAFLD was defined as: 0 for no fatty liver, 1 for mild fatty liver, and 2 for moderate and severe fatty liver. Sex was coded as: 0 for male, 1 for female. HUA is defined as SUA > 420 μmol/L in male, and >360 μmol/L in female. Abnormal AST is defined as AST > 40 IU/L, Abnormal ALT is defined as ALT > 40 IU/L. Low-HDL is defined as HDL-c < 0.90 in men or <1.00 in women. High LDL is defined as LDL-c ≥ 2.6 mmol/L. High TG is defined as TG ≥ 1.7 mmol/L. Central obesity is defined as waist > 90 cm in men and >80 cm in women. HBP is defined as blood pressure ≥ 140/90 mmHg. Age1 is defined as age ≥ 20y and <30y; Age2 is defined as age ≥ 30y and <40y; Age3 is defined as age ≥ 40y and <50y; Age4 is defined as age ≥ 50y and <60y; Age5 is defined as age ≥ 60y

NAFLD was introduced as a dependent variable in the multiple factors logistic regression analysis models (Fig. 3), using sex, hypertension (HBP), central obesity, hyperuricemia (HUA), high-LDL, high-TG, low-HDL, abnormal ALT, and abnormal AST (both classified as yes or no), and age (divided into groups, higher age groups was compared with the lowest age group) as independent variables. In this model, higher age, HBP, central obesity, lipid disorder, and abnormal ALT were risk factors of NAFLD. After adjusting for these risk factors, HUA remained an independent risk factor for NAFLD, and lean-subjects with hyperuricemia had an OR of 1.718 (95% CI 1.622–1.820) to have NAFLD.

**Fig. 4:**
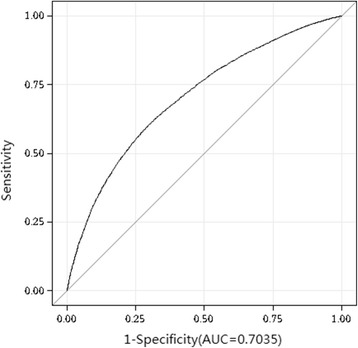
Receivers Operating Characteristic (ROC) Curve for Detecting Mild fatty liver by Ultrasonography Based on the Serum Uric Acid

**Fig. 5:**
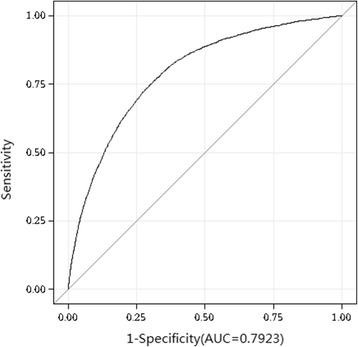
Receivers Operating Characteristic (ROC) Curve for Detecting Moderate and Severe NAFLD by Ultrasonography Based on the Serum Uric Acid

Further, we used a receiver operating characteristic (ROC) curve analysis to evaluate the diagnostic performance of SUA to discriminate the grade of nonalcoholic fatty liver on ultrasonography. The area under curve (AUC) for detecting mild fatty liver based on SUA was 0.70 (optimal cutoff value, 341.05 μmol/L; sensitivity, 70.7%; specificity, 59.9%; Youden index, 0.31); and the AUC for detecting moderate and severe fatty liver based on SUA was 0.78 (optimal cutoff value, 370.15 μmol/L; sensitivity, 73.7%; specificity, 71.2%; Youden index, 0.45). (Figs. 4 and 5).

## Discussion

We observed a positive association between elevated serum uric acid levels and the risks of NAFLD in non-obesity Chinese population. Our data showed the important role of SUA as a crucial independent risk factor for NAFLD. Our findings showed lowest prevalence of NAFLD (8.16%) than other two studies. An earlier Chinese cross-sectional study [7] reported higher prevalence of NAFLD (14.7%). A recent study [15] reported the highest prevalence of NAFLD (27.6%) in non-diabetic Chinese men living in coastal areas have a relatively more seafood diet, which is related with elevated SUA. The possible reason for the different prevalence may due to the characteristics of the studied populations. First of all, our study included only the non-obesity subjects, as we all known they had lower risks of NAFLD. Second, the SUA levels in the coastal areas are higher than in the interior, and so the risks of fatty live will not be the same. Last but not the least; it is easy to see men have higher risks of HUA and NAFLD than women. Evidence from longitudinal studies suggests that the incidence of NAFLD is higher in the male as compared to the female gender. Indeed other longitudinal studies suggest that estrogens are a protective factor for the development of NAFLD. A large body of evidence now definitely supports the notion that the prevalence of NAFLD is higher in men than in women [16]. Also, in our study, we found mild fatty liver in the male accounted for 77.22%, while moderate and severe fatty liver in the male accounted for 86.61%, both which were significantly higher than in female. And, the multi-factor logistic regression analysis showed female gender was an independent protective factor for NAFLD with an OR of 0.40 (95%CI 0.377–0.425).

The epidemiological studies have suggested a positive relationship between SUA and NAFLD; a study [5] of 8925 subjects in East China’s Zhejiang province found hyperuricemia was related to NAFLD, independently of metabolic risk factors at beginning, and after a 3-year follow-up, SUA levels were independently and positively associated with the risk for incident NAFLD, although insulin resistance was not considered. Another study [15] found that in Chinese men, elevated SUA is significantly associated with NAFLD, independent of insulin resistance and other metabolic disorders, such as central obesity or hypertriglyceridemia. Although SUA increase is also observed in individuals with insulin resistance, they found that the increased risks for NAFLD by hyperuricemia could not be explained merely through peripheral insulin resistance.

Obesity, which is an important component of metabolic syndrome (MetS), may play a crucial role in the relationship of SUA and NAFLD [17]. Most of these studies included individuals with obesity, which may have a direct influence on both SUA and NAFLD [10]. Some [15, 18] included individuals without hypertension or diabetes, which is known to have influence on both SUA and NAFLD. There was no population-based study focused on the relationship between lean-NAFLD and SUA levels. Chinese people have their own lifestyle and genetic characteristics, which are different from western population [19]. With the increasing prevalence of NAFLD in lean individuals, it is essential to evaluate the association of lean-NAFLD and SUA levels [20].

So, in the present study, we excluded obese subjects (BMI>25 Kg/m^2^) to analyze the association of SUA and lean-NAFLD. Our data showed positive associations between elevated SUA levels and NAFLD risk in the non-obese Chinese adults, independent of other metabolic factors, such as serum lipid levels, blood pressure, liver enzyme, and so on.

Accumulating clinical evidence suggested that hyperuricemia was significantly associated with NAFLD, which may relate to several underlying mechanisms. Elevated SUA levels may prompt the development of insulin resistance (IR) by reducing endothelial nitric oxide (NO) bioavailability and supply to cells [4]. Beyond hyperinsulinemia, uric acid could originate from fructose metabolism, which is well known for inducing hepatic steatosis being directly metabolized to triglycerides in the liver, and be responsible for mitochondrial oxidative stress [21, 22]. Oxidative stress plays a key-role in steatosis induced by uric acid [23–25]. In conclusion, SUA is able to regulate lipid production and to foster the onset of metabolic disorders and NAFLD through multifaceted pathways. Gu et al. [26] found that deprivation of Slc7a3a would lead to hepatic steatosis in zebra fish as a result of defects in arginine-dependent NO synthesis. And they revealed a NO-AMPK-PPAR-α-signaling pathway that is crucial for the control of hepatic fatty acid oxidation, which might offer new drug targets for clinical treatments of NAFLD.

During the last few years, several parameters evaluated as possible predictors of NAFLD. In fact, increasing evidence shows that SUA levels are associated with NAFLD and even severe liver damage [5, 6]. In this study, we found uric acid levels were used to diagnose fatty liver in different grades with a certain degree of accuracy. As we mentioned before, many researchers worked hard to find a simple, noninvasive and reliable biomarker for follow-up of patients with NAFLD, this study suggested that SUA could be used as a clue to the severity of NAFLD, and it could also be considered as a simple and noninvasive way to follow up patients with NAFLD. However, SUA would not be the sole or the best biomarker. Other predictors, such as platelet count, [27] neutrophil-to-lymphocyte ratio, [28] hyaluronic acid levels, [29] and so on were reported to be associated with the presence of NAFLD. However, some studies [30, 31] found that these associations were not statistically significant. So there is a definite need to look for other noninvasive serum markers for detecting severity of NAFLD.

There are several potential limitations of our study. The main limitation is the lack of information on lifestyle and diet, which may be helpful to understand the relationship between NAFLD and SUA levels. Further studies including detailed personal information were required. Secondly, the diagnosis of NAFLD was based on ultrasonography, with lower sensitivity and specificity versus liver biopsy. Thirdly, most of the subjects included in this study were residents of inland Chongqing, due to different lifestyle and eating habits of inland and coastland residents, the association of lean-NAFLD and SUA levels might not be the same. Further multicenter studies may be helpful.

## Conclusions

In conclusion, our data showed positive associations between elevated SUA levels and lean-NAFLD risk in the inland Chinese adults, independent of other metabolic factors. Our study also suggests that SUA could be used as a clue to the severity of lean-NAFLD, and it could also be considered as a simple and noninvasive marker to follow up patients with lean-NAFLD.

## Abbreviations

ALT: Alanine aminotransferase
AST: Aspartate aminotransferase
AUC: Area under curve
BMI: Body mass index
CI: Confidence interval
DBP: Diastolic blood pressure
GLMs: Generalized linear models
HDL-c: High density lipoprotein-cholesterol
HUA: Hyperuricemia
IR: Insulin resistance
LDL-c: Low-density lipoprotein-cholesterol
MetS: Metabolic syndrome
NO: Endothelial nitric oxide
ORs: Odds ratios
ROC: Receiver operating characteristic
SBP: Systolic blood pressure
SD: Standard deviation
SUA: Serum uric acid
TC: Total cholesterol
TG: Triacylglycerol
